# The Italian Sensorimotor Norms: Perception and action strength measures for 959 words

**DOI:** 10.3758/s13428-022-02004-1

**Published:** 2022-10-28

**Authors:** Claudia Repetto, Claudia Rodella, Francesca Conca, Gaia Chiara Santi, Eleonora Catricalà

**Affiliations:** 1 Deptarment of Psychology, Università Cattolica del Sacro Cuore, Largo Gemelli 1, 20123 Milan, Italy; 2 IRCCS Mondino Neurological Institute, Pavia, Italy; 3 ICoN Cognitive Neuroscience center, Institute for Advanced Studies, IUSS, Pavia, Italy

**Keywords:** Motor system, Sensory system, Semantics, Concepts, Norms

## Abstract

Neuroscience research has provided evidence that semantic information is stored in a distributed brain network involved in sensorimotor and linguistic processing. More specifically, according to the embodied cognition accounts, the representation of concepts is deemed as grounded in our bodily states. For these reasons, normative measures of words should provide relevant information about the extent to which each word embeds perceptual and action properties. In the present study, we collected ratings for 959 Italian nouns and verbs from 398 volunteers, recruited via an online platform. The words were mostly taken from the Italian adaptation of the Affective Norms for English Words (ANEW). A pool of 145 verbs was added to the original set. All the words were rated on 11 sensorimotor dimensions: six perceptual modalities (vision, audition, taste, smell, touch, and interoception) and five effectors (hand-arm, foot-leg, torso, mouth, head). The new verbs were also rated on the ANEW dimensions. Results showed good reliability and consistency with previous studies. Relations between perceptual and motor dimensions are described and interpreted, along with relations between the sensorimotor and the affective dimensions. The currently developed dataset represents an important novelty, as it includes different word classes, i.e., both nouns and verbs, and integrates ratings of both sensorimotor and affective dimensions, along with other psycholinguistic parameters; all features only partially accomplished in previous studies.

## Introduction

Embodied cognition theories have pointed out that conceptual knowledge is grounded in our sensory-motor experience (Wilson, 2002). Concrete concepts, such as *zebra* and *knife*, have been described to be stored in distributed brain areas involved in the sensorimotor processing of the most relevant features of the concept (Catricalà et al., 2015; del Prado et al., 2006; Garagnani & Pulvermüller, 2016; Pulvermüller, 1999; Pulvermüller & Fadiga, 2010). While initial theories claimed a distinction between two types of features, i.e., sensorial and functional (Warrington & Shallice, 1984), recent proposals have highlighted a more fine-grained differentiation. Accordingly, action verbs activate the motor and premotor cortices (Hauk et al., 2004; Tettamanti et al., 2005), while words more heavily endowed with shape (del Prado et al., 2006) and color information (Goldberg et al., 2006) and auditory (Kiefer et al., 2008), olfactory (González et al., 2006), and gustatory properties (Barrós-Loscertales et al., 2012) activate the brain networks involved in the corresponding perceptual processes.

More recently, imaging studies, often associated with computational approaches (Borghesani et al., 2016; Carota et al., 2017), have highlighted that the semantic information is distributed across both modality-preferential sensorimotor and multimodal areas, the latter enabling the integration of motor and sensory information (Fernandino et al., 2016).

A further extension of this framework has interestingly been reported for abstract concepts as well, such as *happiness* and *idea*, although they lack a clear referent that can be experienced through the senses and by means of motor interaction. Abstract concepts have been traditionally described as relying on verbal information (Paivio, 1991), and as less readily connected to contextual details compared to concrete ones (Schwanenflugel & Shoben, 1983). However, grounded cognition approaches (Barsalou, 1999, 2008; Barsalou et al., 2003) posit that, in analogy with concrete concepts, other experiential dimensions are relevant in the definition of abstract concepts (Borghi & Cimatti, 2009; Desai et al., 2018). A central contribution of affective and emotional information has been stressed (Kousta et al., 2011; Vigliocco et al., 2014), involving brain regions known to be associated with affect processing, such as the cingulate cortex (Vigliocco et al., 2014), and the anterior (Conca, Catricalà, et al., 2021b; Wang et al., 2019) and mid-posterior temporal areas (Skipper & Olson, 2014). Similar evidence has been reported for further specific dimensions, such as interoception (Connell et al., 2018; Villani et al., 2021), social (Borghi et al., 2019; Diveica et al., 2022) and quantity-related information (Fischer & Shaki, 2014; Shaki & Fischer, 2008), the latter two in turn respectively involving those brain areas associated with social cognition processing, i.e. superior anterior temporal lobe, and with magnitude, i.e. frontoparietal areas) (Catricalà et al., 2020, 2021; Conca, Borsa, et al., 2021a; Conca, Catricalà, et al., 2021b; Conca & Tettamanti, 2018).

All in all, findings from this line of research have important consequences not only in the domain of semantics but also in cognitive psychology and neuropsychology. Indeed, the setup of experimental studies involving linguistic stimuli, regardless of the specific tasks, requires a thorough pre-screening of the psycholinguistic properties of the stimuli, as they are known to influence linguistic processes (Connell & Lynott, 2012); in addition, the widespread brain network involved in word representation points to the need to consider the different dimensions characterizing each concept. Accordingly, psycholinguistic research should consider a wide range of perceptual, motor, and affective attributes in characterizing both concrete and abstract knowledge, and should offer unified datasets containing ratings for those dimensions. As we will outline in the next section, existing corpora only partially fulfill these needs, thus highlighting the importance of the initiative conducted in the present Italian study.

### Existing corpora investigating sensorimotor dimensions

One of the first attempts to investigate the perceptual dimensions of concepts was made by Lynott and Connell (2009, 2013). They collected ratings on over 800 English words (adjectives, nouns) of perceptual strength, namely how strongly each word is experienced through one of the five perceptual modalities, i.e., visual, haptic, auditory, olfactory, and gustatory. Although these corpora are highly valuable in distinguishing different sensory modalities, they lack the contribution of motor information in defining word meaning. Other studies considered the motor dimension in terms of body–object interaction (BOI), which refers to the degree to which one could physically interact with a word’s referent (Muraki et al., 2022; Siakaluk et al., 2008; Tillotson et al., 2008), or of the relative embodiment dimension (Sidhu et al., 2014; see also Borghi & Cimatti, 2010), which assesses the degree to which the meaning of the word involves the human body. However, they all lack the perceptual dimensions and motor dimension ratings referring to specific body effectors. Amsel et al. (2012) combined ratings of sensory (color, motion, sound, smell, taste, and pain) and motor (graspability) dimensions, which describes a very specific action performed with the hand, leaving the actions performed with other effectors unexplored.

The most complete corpus concerning the sensorimotor dimensions in English is the Lancaster Sensorimotor Norms dataset (Lynott et al., 2020), an English corpus including more than 40,000 words from different linguistic classes, e.g., nouns, verbs, and adjectives, encompassing both perceptual and motor dimensions (considering every single effector separately).

In Italian, until a few years ago, psycholinguistic research was based on corpora including psycholinguistic properties such as concreteness, imageability, and age of acquisition (Barca et al., 2002; Della Rosa et al., 2010), or affective properties such as the affective norms for a large set of Italian words, which assesses valence, arousal, and dominance, together with other classical psycholinguistic properties (familiarity, imageability, and concreteness), and five objective psycholinguistic indexes (word frequency, word class, number of letters, number of orthographic neighbors, mean frequency of orthographic neighbors) (Montefinese et al., 2014).

More recently, new corpora incorporating sensorimotor dimensions have been developed, although not including all the aforementioned variables. For example, Morucci et al. (2019) and Vergallito et al. (2020) collected ratings only of five perceptual modalities, while Villani et al. (2019) rated 425 abstract nouns on 15 dimensions including the perceptual strength based on the five perceptual modalities, the motor dimensions, namely the BOI and the hand- and mouth-related actions, emotionality, metacognition, social metacognition, interoception, and social valence, as well as other classical psycholinguistic features.

### The present study

The present study aims to provide the scientific community with a corpus of linguistic stimuli assessed on different dimensions, ranging from sensorimotor to affective properties, which are still lacking in the available Italian datasets.

To fill this gap, we developed a new Italian database, including the 11 sensorimotor dimensions as in the Lancaster norms. To also include affective dimensions, we capitalized on the affective norms for a large set of Italian words (Montefinese et al., 2014): indeed, we selected the majority of words from that corpus (see Materials and methods section for details), and we added the rating on the new sensorimotor dimensions so that, in the end, each word was evaluated on 17 dimensions (11 sensory-motor dimensions and six affective dimensions).

## Materials and methods

### Participants

A total of 434 unique volunteers, recruited via the online platform Prolific (https://prolific.co/), took part in the survey. Participants were Italian speakers without any language disorders. They were reimbursed £8.50/hour (M duration = 36.79 minutes; SD duration = ±24.51 minutes). Among participants, 391 carried out the main experiment by rating a list of words for the sensorimotor dimensions, while 42 participants completed ratings for the affective dimensions (see below). Table 1 summarizes participants’ characteristics for age, sex, and education. The study was approved by the Ethics Committee of the Catholic University of the Sacred Heart of Milan, Italy.

**Table 1:** Participants’ demographic characteristics

*Sex*	*N*	*Age M, (SD)*	*Education level (N, percentage)*
*Male*	226	25.9 (6.9)	1 (10, 4.4%); 2 (119, 52.7%); 3 (1, 0.4%); 4 (70, 31.0%); 5 (16, 7.5%); 6 (7, 3.1%); 7 (3, 1.3%)
*Female*	198	28.6 (10.7)	1 (1, 0.5%); 2 (100, 50.5%); 3 (4, 2.0%); 4 (37, 18.7%); 5 (39, 19.7%); 6 (11, 5.6%); 7 (6, 3.0%)
*Other*	9	23.9 (3.2)	1 (1, 11.1%); 2 (6, 66.7%); 3 (0, 0%); 4 (1, 11.1%); 5 (1, 11.1%); 6 (0, 0%); 7 (0, 0%)

### Materials

Our dataset consists of 959 Italian words, including 759 nouns and 200 verbs in the infinitive form, spanning the entire range of concreteness (5 being the intermediate value of the Likert scale, 279 words rated < 5, and 680 words rated > 5 for concreteness). Most of the items (814 words; 84.9%) were taken from the work by Montefinese et al. (2014), while the other 24.1% (145 words, all verbs) were taken from other studies (Dalla Volta et al., 2009; Papeo et al., 2010, 2011; Repetto et al., 2015) and were added to increase the number of verbs in our dataset.

### Data collection: Sensorimotor norms

We randomly divided the dataset into 19 lists of 50 words (except for one list, which consisted of 49 words). We additionally included the same 10 control words, comprising both nouns and verbs [*acqua* (water), *agilità* (agility), *calciare* (to kick), *cancellare* (to delete), *distruggere* (to destroy), *fanghiglia* (slush), *grattacielo* (skyscraper), *ignoranza* (ignorance), *negare* (to deny), *umiliare* (to humiliate)], in every list to assess the level of agreement between evaluators.

**Fig. 1:**
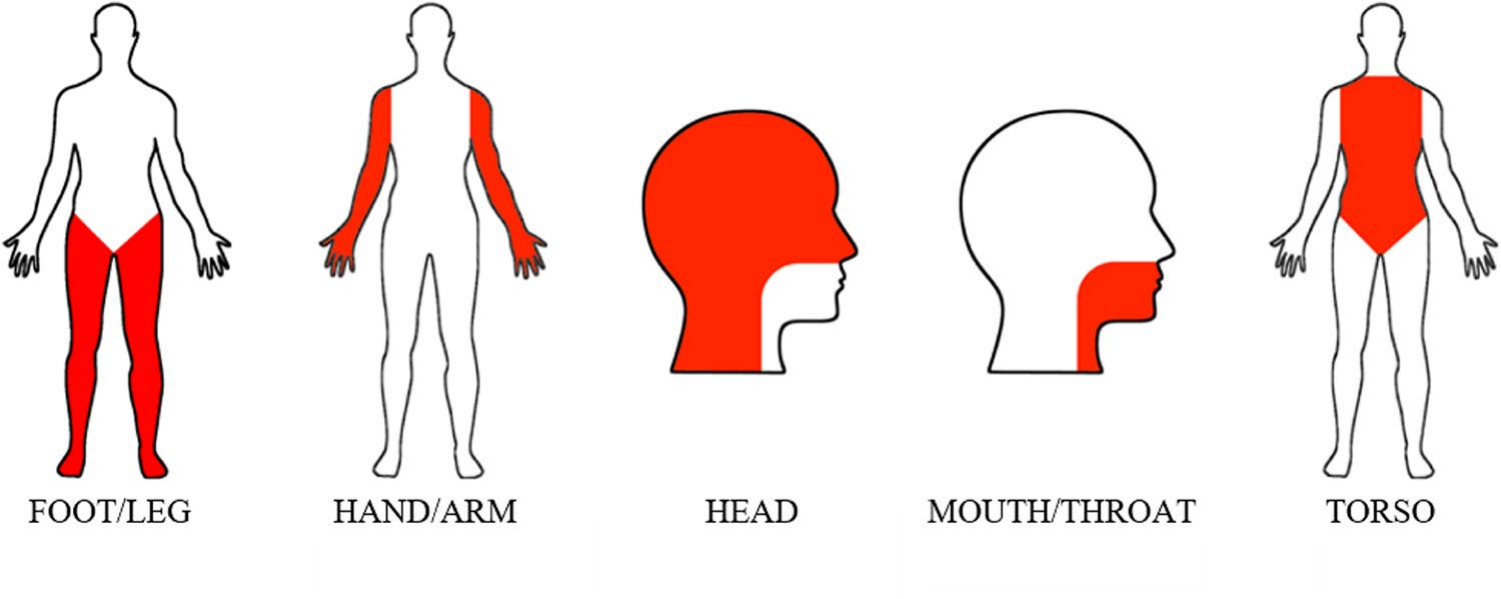
The five different body parts showing the action effectors, taken from Lynott and collaborators (2020)

Each participant rated one list of 60 words (i.e., 50 words + 10 control words), and each item was thus rated by a mean of 20.37 participants (SD = 0.84). For each word list, participants rated all the modalities of perceptual strength and all the effectors of action strength, i.e., 11 dimensions in total. The data collection was conducted online using Qualtrics survey software (https://www.qualtrics.com). After reading the information sheet and consenting to take part in the research, participants were asked to rate the list of words. They received detailed instructions about the scales they should use to evaluate each word; in addition, an example of a possible rating was provided using a word that was not included in our set of items. The example presented a possible answer, but the absence of right or wrong responses was stressed. In line with this, we did not give participants explicit instructions about ambiguous words. Following the procedure developed by Lynott et al. (2020), participants were asked to rate how much they experienced the concepts using six perceptual senses and five action effectors from different body parts. The perceptual modalities were touch, hearing, smell, taste, vision, and interoception (i.e., sensations inside the body). The action effectors were mouth/throat, hand/arm, foot/leg, head (excluding mouth/throat), and torso. The rating scales ranged from 0 (not experienced at all through that sense/action) to 5 (highly experienced through that sense/action). Participants could skip to the next word if they did not know the meaning of a word or if they preferred not to answer, by clicking the corresponding box (i.e., “I do not know the meaning of this word” or “I prefer not to answer”). The five body parts were shown to the participants through a picture in which each effector was highlighted (Lynott et al., 2020) (Fig. 1). The instructions were as follows: “How much do you experience WORD through an action of *mouth/throat, hand/arm, foot/leg, head excluding mouth/throat, and torso*?”; “How much do you experience WORD by *feeling through touch, hearing, sensations inside your body, smelling, tasting*?”, where WORD was replaced with each term of the list^1^.

Participants took about 36 minutes to complete the entire experiment (M = 36.28 minutes; SD = ±23.57 minutes).

### Data collection: Affective norms, familiarity, imageability, and concreteness

Additional data were collected to complete our dataset with the affective norms for the 144 verbs that we added. Namely, valence (i.e., the extent to which a given word refers to something positive or negative), arousal (i.e., the extent to which a given word refers to something arousing or calm), dominance (i.e., the extent to which a given word refers to something submissive or dominant) [(i.e., Self-Assessment Manikin, SAM) (Bradley & Lang, 1994, 1999)] and familiarity (i.e., frequency with which a given word is presented in everyday life, in both written and spoken form), imageability (i.e., the extent to which a word can recall a mental image, a figure, a sound, or a perceptual sensation), and concreteness (i.e., the extent to which a word denotes something that can be perceived directly by the senses) (FIC) were rated following the procedure adopted by Montefinese et al. (2014). Rating scales ranged from 1 (very unpleasant, very calm, very submissive, unfamiliar, unimaginable, abstract) to 9 (very pleasant, very aroused, very dominant, highly familiar, highly imaginable, highly concrete, respectively).

Participants were asked to rate a list of 72 verbs with regard to FIC and SAM scales, and took about 35 minutes to complete the task (M duration = 35.53 minutes; SD duration = ±17 minutes).

*N* number, *M* mean, *SD* standard deviation; education level: 1 = primary school, 2 = high school, 3 = professional school, 4 = bachelor’s degree, 5 = master’s degree, 6 = prost-graduate school, 7 = PhD

### Data quality check

We included three levels of attention checks. In the first, instructions were replaced with “Please select 3 for every effector/sense”; thus, participants had to select a given response, namely 3, on the scale. This helped to identify those participants who answered without paying attention. Seven participants were excluded as they failed at least one attention check. A second quality check consisted of controlling how many participants gave the same answer in 85% or more of the questions. None of them was excluded following this criterion. Finally, responses that were at least three SDs above or below the dimension mean were considered outliers (1.5% of our data) and were not included in the analysis.

### Data analysis

Means and standard deviations per dimension per word were calculated. To account for the interrater reliability, we calculated the mean Cronbach’s alpha per item list and then averaged for each dimension. Moreover, Cronbach’s alpha was calculated for the 10 control words per dimension to control for the degree of agreement in the entire sample and to verify whether the subsamples that completed the different lists of words were comparable.

#### Dominance and exclusivity scores

To analyze the degree of dominance through all the dimensions, we firstly identified the dimension with the maximum rating for each item, considering separately the perceptual modality, the action effector, and then taking into account the overall sensorimotor dimensions.

The exclusivity score (ES), namely the extent to which each concept is experienced through a specific dimension, was also calculated. This index gave us information about the multidimensionality of each concept: if a word was rated equally in more than one dimension, it means that it could be experienced through different perceptual modalities and/or through the action of different body parts. The exclusivity score ranged from 0 (completely multidimensional) to 1 (completely unidimensional and experienced through a single specific dimension), and it was calculated as the ratio between the rating range and the sum of the scores obtained in the different dimensions (Lynott & Connell, 2009).

#### Correlation analysis

To investigate the relations between different dimensions, we conducted exploratory Pearson’s correlation analyses: (a) between the sensorimotor dimensions and (b) between the sensorimotor and affective dimensions.

#### Principal component analysis

To reduce the complexity of our data, highlighting possible trends and clusters, we ran two different principal component analyses (PCA), both with varimax rotation with Kaiser normalization. The first one was in order to observe the distinctness of information captured by the 11 sensorimotor dimensions, while the second PCA considered sensorimotor as well as affective variables. To test the adequacy of the data for PCA, we calculated the Bartlett test of sphericity and the Kaiser-Meyer-Olkin measure of sampling adequacy (MSA). We considered the eigenvalue (>1), the scree plot, and the total variance explained to identify the number of factors.

Uniqueness values were reported for each effector and perceptual modality, indicating the proportions of unique variance, i.e., variance not shared with other dimensions.

## Results and discussion

**Fig. 2:**
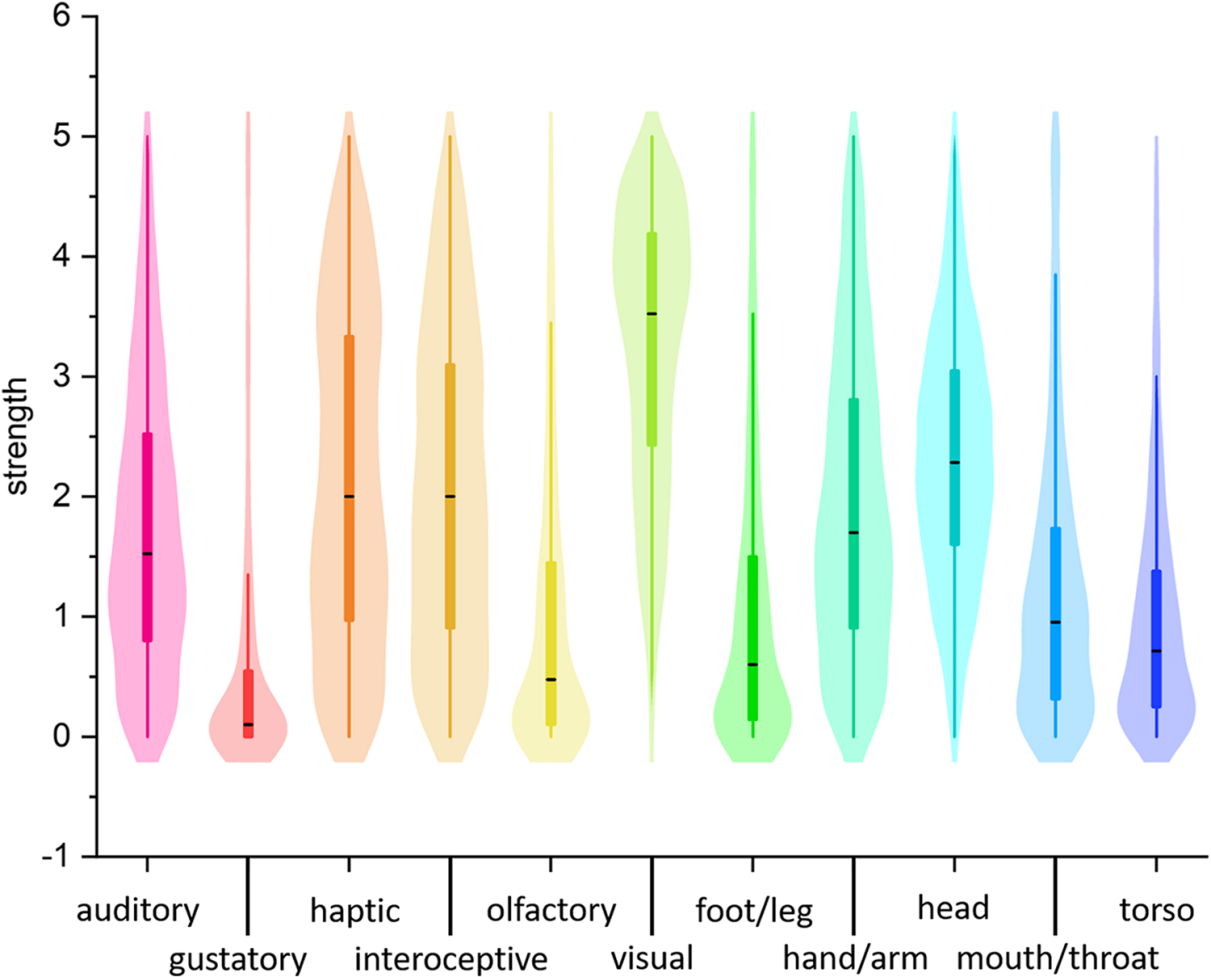
Distribution of sensorimotor strength ratings of the 11 dimensions; black lines indicate the median value of each dimension

A new Italian database was created containing the mean and standard deviations for each of the 11 dimensions per 959 words (Fig. 2). One hundred and ninety participants did not rate at least one word, as they did not know the meaning (135 cases), or as they preferred not to answer (155 cases). A total of 90.8% of the words were rated by at least 20 participants, and only 10 concepts were evaluated by less than 18 individuals (e.g., *scorbuto*, scurvy). Among the 10 control words, the one that obtained the lowest number of ratings was *fanghiglia* (slush), which was evaluated by 351 subjects (88% of the entire sample).

Results showed excellent reliability across raters for each perceptual modality (Cronbach’s alpha: auditory .93, gustatory .97, haptic .95, interoceptive .94, olfactory .96, visual .92) and effector (Cronbach’s alpha: foot .95, hand .94, head .87, mouth .94, and torso .92). In addition, from the analysis of the 10 control words, we found an extremely high level of agreement in the whole sample, as Cronbach’s alpha ranged from 0.99 to 1.00 for all the dimensions. In other words, the 10 control words were evaluated in a very consistent way by all the raters.

**Fig. 3:**
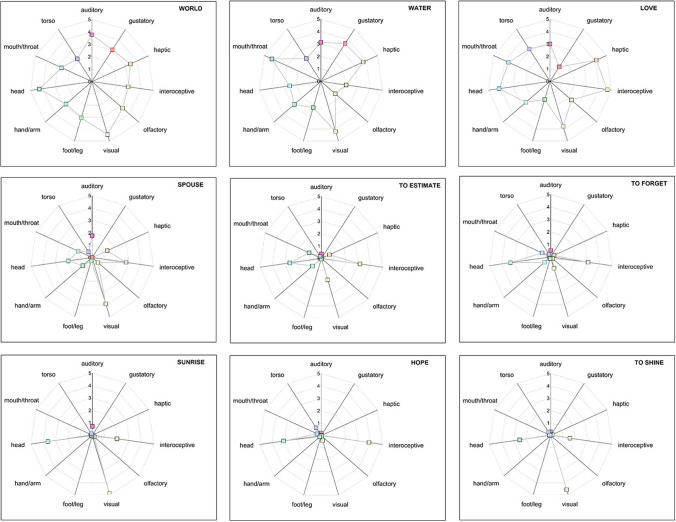
Polar plots indicating mean values of the 11 sensorimotor dimensions for 9 representative words (i.e., world, water, love, spouse, to estimate, to forget, sunrise, hope, to shine). The top row displays words that are highly multidimensional, with medium-to-high ratings on several modalities. The bottom row displays words polarized on few dimensions, with low/null scores on the other ones. Middle row displays words with intermediate ratings on multiple dimensions

A summary of the mean sensorimotor strength ratings (0–5) and relative standard deviations for each perceptual modality and effector is shown in Table 2. Vision and head-action received the highest ratings (M = 3.27, M = 2.31, respectively; see also Fig. 2). Figure 3 shows some examples of the distribution of the sensorimotor dimensions for nine different words.

**Table 2:** Summary of the sensorimotor strength ratings, indicating mean (M), standard deviations (SD), and uniqueness value for each perceptual modality and motor effector

Sensorimotor dimension	*M*	*SD*	Uniqueness
Perceptual modalities			
Auditory	1.73	1.18	0.16
Gustatory	0.54	1.02	0.12
Haptic	2.12	1.38	0.20
Interoceptive	2.08	1.31	0.18
Olfactory	0.96	1.14	0.36
Visual	3.27	1.14	0.31
Motor effectors			
Foot/leg	1.02	1.16	0.35
Hand/arm	1.91	1.25	0.33
Head	2.31	1.02	0.37
Mouth/throat	1.23	1.15	0.24
Torso	0.96	0.94	0.18

### Dominance and exclusivity scores

Results of the dominance scores (see Table 3) suggested that, among the effectors, the head was most commonly perceived as the dominant part of the body, rated as the dominant dimension in 68% of concepts, while the torso was rated as dominant in only 5% of the concepts. When we examined the perceptual modalities, vision was the most dominant dimension (61%), followed by interoception, (38%) and haptic (16%), while smell was the least dominant (4%). Considering the sensorimotor variables together, vision was the most dominant dimension (54%), while movement of the torso was the least dominant (1%). Our results were in line with previous works, suggesting a striking dominance of the visual modality (Lynott et al., 2020; Morucci et al., 2019; Vergallito et al., 2020), and reporting smell and torso as the least dominant dimensions for sensory and motor modalities, respectively (Lynott et al., 2020).

**Table 3:**
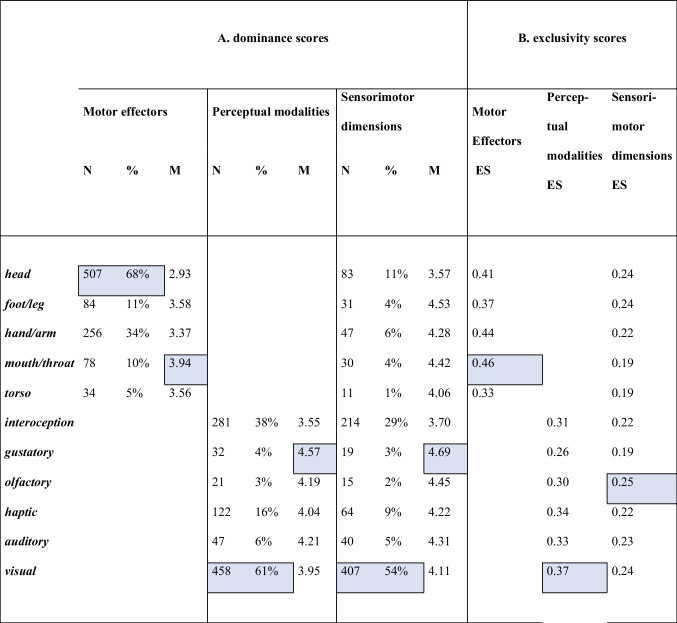
(A) Number (*N*) and percentage (%) of words for which a given effector and/or perceptual modality is the dominant dimension, with the corresponding mean score (M); (B) mean exclusivity scores (ES) for effector and/or perceptual modalities (highlighted boxes indicate the highest value for each index)

**Fig. 4:**
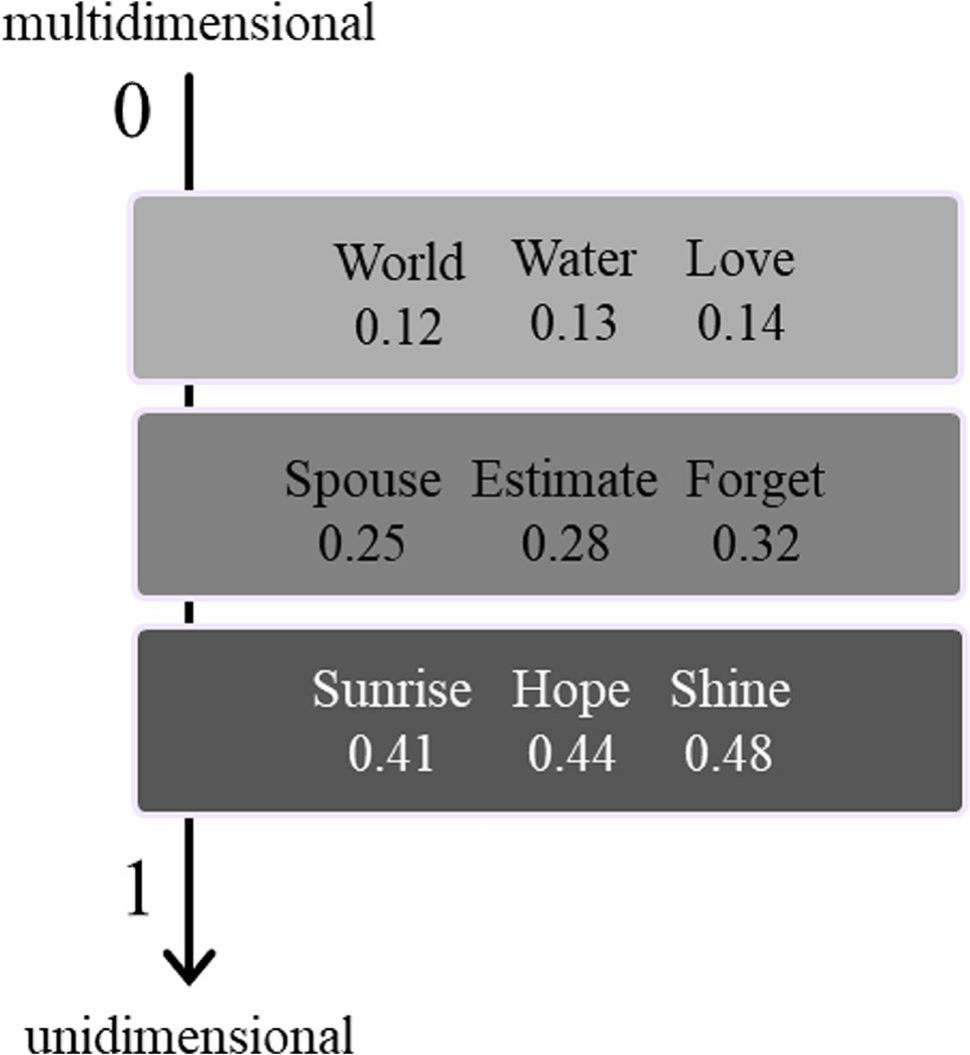
Examples of sensorimotor exclusivity scores (ES) for some of the concepts, from highly multidimensional to highly unidimensional

The summary of the exclusivity scores (ESs) of the 11 dimensions is shown in Table 3. We analyzed perceptual modalities and action components first together (sensorimotor ES) and then separately (perceptual ES and action ES). In line with a previous study by Vergallito and co-workers (2020), we found that smell reached the highest sensorimotor ES (i.e., 0.25), while taste was the most multimodal dimension, together with the action of mouth/throat and torso (ES = 0.19). Overall, the exclusivity score for the 11 sensorimotor dimensions suggested that the concepts were highly multidimensional [sensorimotor ES: M = 0.23; SD = ±0.07, very similar to the correspondent value found in the Lancaster dataset (Lynott et al., 2020), i.e., 0.24], namely experienced through a wide range of perceptual modalities (perceptual ES: M = 0.34; SD = ±0.09), and tended to involve more than one effector (action ES: M = 0.41; SD = ±0.19). In our dataset, the most unidimensional visual concept was *arcobaleno* (rainbow) (ES = 0.58), while the most multidimensional word was *vita* (life) (ES = 0.11), where all the 11 sensorimotor dimensions were involved (rating range: 2.89–4.19). Additional examples of concepts, from highly unidimensional to highly multidimensional, are reported in Fig. 4 (see also Fig. 3 for polar plots of the corresponding means sensorimotor strengths).

### Correlations analyses: (a) Sensorimotor dimensions

Table 4 shows the correlations matrix between the 11 sensorimotor dimensions. Replicating English sensorimotor norms (Lynott et al., 2020), the action of the foot/leg was highly correlated with action of the torso (*r* = .615), presumably because all those concepts share those effectors, like *correre* (to run) or *divano* (sofa), or polarizing on different action modalities, such as mouth/throat as for *bacio* (kiss; action ES = .51; M for mouth/throat = 4.9). In addition, movements of the foot/leg and the torso were positively correlated with action of the hand/arm (foot/leg and hand/arm: *r* = .431; torso and hand/arm; *r* = .504). These correlations may be ascribable to full-body physical activities, such as *lotta* (fight) or *atletica* (athletics). Moreover, a positive correlation was found between the action of the head and the mouth/throat (*r* = .469), likely associated with objects or gestures of eating/drinking, as *acqua* (water), or greatly localized in the zone of the head, as *dentista* (dentist).

**Table 4:**
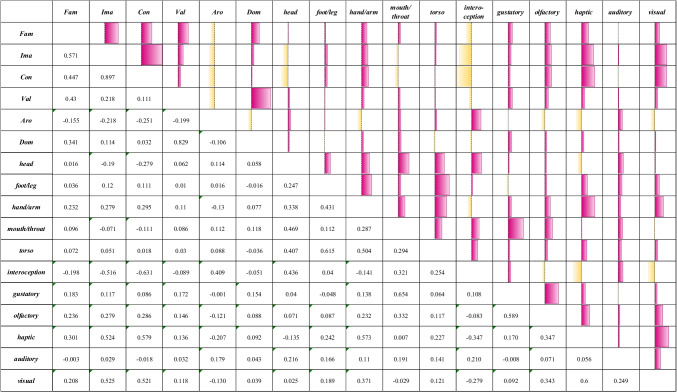
Correlations between sensorimotor and affective dimensions; Fam = familiarity, Ima = imageability, Con = concreteness, Val = valence, Aro = arousal, Dom = dominance; positive and negative correlations are indicated in pink and yellow shades, respectively, and larger shapes represented stronger correlations

Observing the relationships among the perceptual modalities, a negative correlation was found between interoception and touch (*r* = −.347) and interoception and vision (*r* = −.279). Indeed, concepts with higher ratings for interoception could be linked to internal states which were typically not touchable or visible, such as *beatitudine* (bliss; M for concreteness = 3.2); on the other hand, those with higher ratings for touch or vision were associated with concrete concepts that were not linked to a specific internal state, like *cappello* (hat; M for concreteness = 8.5) or *armadio* (wardrobe; M for concreteness = 8.3). Likewise, words that were more related to internal states obtained lower ratings for touch and higher score for interoception, like *aborto* (abortion, M for interoception = 4.0, M for touch 1.78) or *infatuazione* (infatuation, M for interoception = 4.70, M for touch 1.33). Moreover, correlations were observed between taste and smell (*r* = .589), and between touch and vision (*r* = .600), in line with other studies on Italian words (Morucci et al., 2019; Vergallito et al., 2020). As regards the first one, the relationship could be associated with all objects and actions related to food, like *cucinare* (to cook) or *latte* (milk). The second one could instead be explained by concepts defined by things and actions that could be seen and touched, like *albero* (tree) or *evidenziatore* (highlighter). Finally, audition was unrelated to all the other perceptual modalities but interoception, to which it was positively correlated, as in the Lancaster corpus (Lynott et al., 2020). Such a relationship could be identified for concepts like *allerta* (alert) or *canzone* (song), which could trigger internal reactions and are also often denoted by an acoustic feature.

Importantly, as in the study by Lynott et al. (2020), there were also some strong relationships between effectors and perceptual modalities. A positive correlation was indeed encountered between the head and interoception (*r* = .436), plausibly reflecting the association of the head with the activity of the mind, in terms of thinking and cognitive processing in general, as for words like *accettazione* (acceptance) or *intelletto* (intellect). Additionally, not surprisingly, a robust positive correlation was observed between taste and action of mouth/throat (*r* = .654), likely associated with food, and the activities of eating or drinking, as is the case for words such as *torta* (cake) or *vino* (wine). Importantly, the two dimensions were not completely overlapping, because there were concepts that belonged to either one or the other, such as *urlo* (scream), which was certainly related to the action of the mouth (action ES = .51; M for mouth/throat = 4.43), but did not have any gustatory features. Another correlation was found between touch and action of the hand/arm (*r* = .573), probably due to words like *abbraccio* (hug) or *ombrello* (umbrella). Again, this relationship was not always present, for instance in the case of those gestures that involved the hand/arm but did not necessarily concern touch, like *salutare* (to greet). Finally, the positive correlation between action of the hand/arm and vision (*r* = .371) could be associated with manipulable concrete objects, e.g., *ago* (needle; M for concreteness = 8.3) and *bambola* (doll; M for concreteness = 8.2).

In addition, we checked the consistency between our data and those collected by Villani and colleagues, as both datasets share the greatest number of overlapping dimensions (i.e., perceptual modalities: vision, touch, audition, olfaction, gustation; interoception; action modalities: hand, mouth). To this purpose, we selected the shared words between the two datasets (*n* = 106), and we performed Pearson correlations on the ratings gathered on the overlapping dimensions. Results indicated that the ratings were positively correlated on the perceptual dimensions (vision: *r* = 0.693; *p* < 0.001; audition: *r* = 0.706; *p* < 0.001; touch: *r* = 0.737; *p* < 0.001; olfaction: *r* = 0.716; *p* < 0.001; gustation: *r* = 0.673; *p* < 0.001) and on interoception (*r* = 0.756; *p* < 0.001), but not correlated on the action dimensions (hand: *r* = −0.09; mouth: *r* = −0.135). One possible explanation refers to the instructions provided to the raters: whereas for the perceptual dimensions in both studies the (translation of the) wording reported by Lynott and collaborators (2020) was used, for the action modalities the question differed slightly between the two studies (in the present study: “*How much do you experience WORD through an action of*”—with a focus on the subjective experience with the concept; in the Villani study: “*to what extent the concept involves use of”*—with a focus on the actual involvement of the body part), and this could have led to different answers.

### Correlations analyses: (b) Sensorimotor and affective dimensions

As shown in Table 4, there are also interesting relationships between the sensorimotor and the affective dimensions. The dimension of concreteness seemed to represent a key node, as many concrete concepts were indeed visible and touchable. Accordingly, positive correlations were found between concreteness and vision (*r* = .521) and touch (*r* = .579), and also between the latter two dimensions (*r* = .600).

Moreover, concreteness was negatively correlated with interoception (*r* = −.631), thus suggesting that interoception was associated with internal states, emotion, and abstract concepts in general, such as *estasi* (ecstasy) or *fantasia* (fantasy). Interoception was also negatively correlated with imageability (*r* = −.516), i.e., the concepts highly imaginable were less experienced through internal sensations, like *abete* (fir) or *gufo* (owl). Observing the SAM scale (i.e., valence, arousal, dominance), a relationship was found between arousal and interoception (*r* = .409), probably because internal sensations were typically highly stimulating and arousing, as *agonia* (agony) or *passione* (passion). Some positive correlations were found between familiarity and smell (*r* = .236), touch (*r* = .301), vision (*r* = .208), and taste (*r* = .183) and the action of the hand/arm (*r* = .232), suggesting that concrete words, particularly those that can be touched, were also more familiar. Only hearing did not correlate with familiarity, but it was related to arousal (*r* = .179), maybe for all those concepts associated with loud noises, or sounds that provoked strong emotional reactions, alarm, or fear, like *bomba* (bomb) or *strillo* (shriek). Finally, vision correlated with all the other variables, with the exception of dominance (familiarity: *r* = .208; imageability: *r* = .525; concreteness: *r* = .521; valence: *r* = .118; arousal: *r* = −.130). It was not surprising that concepts experienced through vision were also more imaginable and concrete, like *abito* (dress) or *cucire* (to sew). Words that were strongly related to one of the other senses represented the exceptions, like *puzza* (stink; perceptual ES = .61; M for smell = 5) or *calore* (warmth; perceptual ES = 0.35; M for touch = 3.33).

### Principal component analysis

PCA on sensorimotor variables had an acceptable sample size (MSA = .644), and the Bartlett test indicated that the correlations among the variables were sufficiently large to apply this analysis to our data (*p* < 0.0001). Looking at the scree plot and the total variance explained, we decided to extract four components, which in combination explained 74.5% of the variance. The sensorimotor strength ratings (0–5) and uniqueness scores (0–1) are shown in Table 2. Uniqueness indicates the proportions of unique variance (i.e., variance not shared with other components) for each effector and perceptual modality. In line with results obtained by Lynott et al. (2020), taste showed the lowest degree of uniqueness (12.3%), while head action strength had the highest uniqueness score (36.7%) (see table 2). The outlined factors reflected findings previously encountered in correlation analyses. Accordingly, the first factor included the movement of the hand/arm, vision, and touch, along with negative loadings for the action of the head and interoception. The second factor consisted of the action of the mouth and throat, taste, and smell. A third factor included the movement of the foot/leg and the torso. Finally, the fourth factor was composed only of hearing. These data were consistent with those by Morucci et al. (2019), who, considering only the perceptual dimensions, identified three clusters including vision–touch, taste–smell, and hearing, indicating that this structure in semantics holds for different classes of words (specifically, Morucci and coworkers included only adjectives).

In the second PCA, we included the affective variables obtained from the Montefinese database (Montefinese et al., 2014). The Bartlett test of sphericity (*p* < 0.0001) and the MSA (0.70) allowed us to apply the analysis to our data. The Scree Plot suggested extracting five components, which explained 70.6% of the variance. Results showed that Valence was the variable with the lower uniqueness score (11.7%), while Arousal reported the highest degree of uniqueness (50.6%). The five factors identified consisted of: (1) high scores in concreteness, imageability, touch, vision, and low scores in interoception; (2) high scores for the action of the torso, the foot/leg, the hand/arm, and the head; (3) high scores for taste, smell and movement of the mouth/throat; (4) high scores for valence, dominance, and familiarity; (5) high scores for hearing and arousal.

## Conclusions

The Italian Sensorimotor norms dataset includes ratings of sensorimotor strength for 959 Italian nouns and verbs. The pool of words was derived from the Italian adaptation of the Affective Norms for English Words (ANEW) (Montefinese et al., 2014), which was improved with additional verbs, as this word class was underrepresented in the original dataset. The new verbs were also rated for the affective and semantic dimensions included in the Montefinese corpus so that we obtained a dataset of words validated for a great number of semantic dimensions and psycholinguistic indexes: six perceptual modalities, five action effectors, three affective dimensions, familiarity, imageability, concreteness, and five objective psycholinguistic parameters. The norms demonstrated good reliability, as a strong agreement among raters was detected across all the dimensions. In addition, our results are very consistent with other existing databases. To our knowledge, this is the first corpus for the Italian language trying to tap into such a range of different semantic features. Compared to other similar works (Morucci et al., 2019; Vergallito et al., 2020; Villani et al., 2019), the current norms offer some advantages over existing datasets: first, it includes diverse word classes, namely nouns and verbs, which are the most used stimuli in linguistic tasks; second, it spans from perceptual to action strength dimensions, covering the entire set of bodily-based representations as proposed by embodied cognition accounts (Barsalou, 1999, 2008); third, capitalizing on the previous work by Montefinese et al. (2014), it offers the scientific community a new integrated tool for cognitive and linguistic research where every single word is rated along multiple dimensions relevant for catching the complexity of semantics. Indeed, the sensorimotor dimensions have already been successfully applied in psycholinguistic research in different languages. In English, Connell and Lynott (2012) demonstrated that the maximum perceptual strength was the best predictor of word processing performance, surpassing concreteness and imageability. In addition, the same authors (Lynott & Connell, 2013) showed a systematic association between the strength of perceptual experience associated with the concept and its surface word form, suggesting that distinctive perceptual experience tends to draw distinctive lexical labels. Similarly, in Italian, Vergallito et al. (2020) found that the five perceptual modalities accounted for the greatest variance in predicting word reading performance, compared to imageability and to an optimized set of perceptual dimensions including all of them but haptic modality.

In conclusion, we believe that the present work could be of great value in offering a valuable dataset for the construction of experimental studies, with a potential cross-linguistical application.

## Data Availability

The datasets generated during and/or analyzed during the current study are available in the Open Science Framework repository, https://osf.io/rcsnm/.

## Footnotes

1 The exact wordings in Italian were “*In che misura fa esperienza di XXX eseguendo un’azione con*” for the action-related dimensions, and “*In che misura percepisce XXX attraverso*” for the sensory-related dimensions
